# The impact of early breaking bad news education: a qualitative study into students' self-reported take-home messages

**DOI:** 10.15694/mep.2020.000025.1

**Published:** 2020-02-07

**Authors:** Marianne Brouwers, Anne De la Croix, Roland Laan, Chris Van Weel, Evelyn Van Weel-Baumgarten

**Affiliations:** 1 Radboud Institute for Health Sciences; 2 LEARN! Academy; 3 Health Academy

**Keywords:** communication, education, patient centredness, breaking bad news, undergraduate.

## Abstract

This article was migrated. The article was marked as recommended.

**Introduction**: This study investigated the self-reported take-home messages of medical students after an early training module in breaking bad news (BBN). The findings will aid the discussion on how to teach BBN.

**Methods**:We asked 592 second year medical students at two Dutch medical schools to write down what they had learned, right after their first communication skills training in BBN. We analysed these self-reported take-home messages using a hybrid form of qualitative content analysis using SPIKES as a conceptual frame.

**Results**:The most important take-home messages reported by students in both schools were: how to inform the patient (24,5%), how to deal with emotions (20,6%), and how to prepare for a BBN-consultation (16,9%). Additionally, students reported the professional quality “being patient-centered”.

**Conclusions**:Early communication skills training on BBN, including true stories and examples of doctors and their BBN-experiences, does not only give the students the knowledge how to break bad news to patients, but also which underlying professional qualities are needed to successfully break bad news. Good role models and realistic examples are therefore important. We propose a new mnemonic PRINSE (PReparation-INformation-Silence-Emotions) for novice learners in the design of a helical curriculum.

## Introduction

Breaking bad news (BBN) is often a difficult, but important everyday task for health professionals, and particularly doctors ( Ptacek and Eberhardt, 1996; Friedrichsen and Milberg, 2006; van Dulmen *et al.*, 2007). Bad news is considered any information that negatively changes a person’s expectation about the present and the future, and is highly patient dependent ( Buckman, 1984; Minichiello, Ling and Ucci, 2007). Poorly communicated bad news affects both the patient and the doctor: patients can experience increased stress and anxiety and show poor adjustment to the bad news with reduced health outcomes when it is not been told properly ( Baile *et al.*, 2000; VandeKieft, 2001). Additionally, research suggests that an inappropriate BBN conversation is not only negative for the patient, doctors can also experience stress related to poorly broken bad news, which can contribute to burn-out and anxiety ( Ramirez *et al.*,1995).

The relevance of an adequate bad news consultation is clear. But what is the right way to break bad news? Over the years several useful models, such as SPIKES (Setting up interview - assessing Perception patient - obtaining patient’s Invitation - giving Knowledge and information - addressing Emotions - Strategy and summary) and ABCDE (Advance preparation - Build a therapeutic environment - Communicate well - Deal with patient and family reaction - Encourage and validate emotions), and guidelines on BBN have been developed ( Girgis and Sanson-Fisher, 1995; Baile *et al.*, 2000; Ambuel and Mazzone, 2001; Minichiello, Ling and Ucci, 2007; Narayanan, Bista and Koshy, 2010). Nonetheless, (future) doctors report that their medical school did not prepare them for dealing with important bad news topics, such as death and dying ( Brennan *et al.*, 2010; Gibbins, McCoubrie and Forbes, 2011), while their actual performance in BBN leaves much to be desired for ( Eggly *et al.*, 1997). Also, medical students ( Toivonen *et al.*, 2017) and experienced physicians ( Shaw *et al.*, 2013) experience strong and perplexing feelings when confronted with BBN. This stresses the importance of appropriate education and skills training in BNN ( Rosenbaum, Ferguson and Lobas, 2004).

Clinical communication skills training has become an important part of medical training, since adequate and effective doctor-patient communication training is essential to provide good care. Berkhof *et al*. ( Berkhof *et al.*, 2011) showed that effective communication skills training programs should be active and practice-oriented (including roleplay, feedback and small group discussions). Breaking bad news depends heavily on communication skills, but also requires an in-depth orientation on the clinical content. Knowledge and skills about how to break bad news can be acquired through various educational approaches, varying from lectures to real life patient contact. Teaching about BBN is shown to have an impact on doctor’s self-confidence ( Rosenbaum, Ferguson and Lobas, 2004). Also, performance ( Colletti *et al.*, 2001; Gorniewicz *et al.*, 2017) and self-perceived competence ( Schildmann *et al.*, 2006) increased after BBN skills training. Residents found simulation the most helpful in order to learn how to BBN ( Park *et al.*, 2010). However, lectures are shown to be the most common tool when teaching end-of-life care, including breaking bad news, since they take minimal time and faculty resources to deliver information to a large audience ( Billings and Block, 1997; Fitzpatrick *et al.*, 2017). At best, this approach transfers knowledge but not skills as it does not allow practice, feedback and refinement of skills. To address skills, Lamba *et al*. ( Lamba *et al.*, 2016) stressed that the modality of choice for BBN training for surgery residents nowadays is simulation.

Additionally, there is growing evidence that student achievement (knowledge, skills, attendance and student engagement) is strongly associated with the level of interaction in education ( Deslauriers, Schelew and Wieman, 2011). A few variables associated with good quality of classroom interaction are: time for discussion and questions, small group sizes, in-class tasks for students, and active student participation ( Schneider and Preckel, 2017). These findings are in line with another review that shows that the most effective approach to teaching and training BBN includes presenting basic steps to effectively delivering bad news, and gives learners the appropriate time to discuss concerns, practice, and receive feedback on their skills ( Rosenbaum, Ferguson and Lobas, 2004). In addition, it should be provided early and often and gives students a framework in which role models, when giving bad news, can be critically evaluated ( Rosenbaum, Ferguson and Lobas, 2004). Furthermore, it is suggested that communication skills are best learned when they are reiterated and reinforced throughout medical training longitudinally building on each previous step (helical model) ( Kurtz, 1998; Silverman, 2009). Ideally, such a helical model starts with introducing theory about BBN, followed by skills training in a controlled environment and finally clinical practice, as suggested by leading theories ( Kaufman, 2003).

In short, approaches in offering bad news education to medical students vary widely, and the evidence shows that starting early and continuing to build on this first experience, with a combination of knowledge transfer and experiential learning with practice and feedback is best practice. However, models and guidelines on BBN are developed for doctors with clinical experience and include steps regarding follow-up and treatment plan and do not meet the needs and level of students and are less useful for training BBN at this stage of their career. In order to build an effective helical curriculum, it is necessary that novice students first learn the basic steps in an as simple as possible way based on prior knowledge and experience and that this training is just-in -time. Therefore, the aim of our study was to gain insight into what novice students pick up after a first introduction to BBN. This knowledge will act as a starting point to revise the current helical curriculum on BBN, that develops into the full comprehensive scope of a BBN conversation at the end of their programme. Our research question was: what themes can be identified from students’ take-home messages after a first introduction to BBN?

Although we collected the data in 2011, a Pubmed search (2019) convinced us that since 2011 no articles had been published regarding this subject.

## Methods

### Study context

The study was carried out at the Radboud University Medical Center in Nijmegen (school 1) and at the Erasmus Medical Center in Rotterdam (school 2) in fall/winter 2010. Both schools have a helical curriculum on BBN skills training, consisting of multiple sessions throughout the medical course (a six-year Bachelor/Master curriculum) ( van Weel-Baumgarten *et al.*, 2012), as integral part of the clinical communication skills training curriculum ( table 1).

**Table 1.** Description of the helical curriculum on BBN skills training at school 1 and 2.

Medical school	BACHELOR				MASTER			
	Yr 1	Yr 2		Yr 3	Yr 4	Yr 5	Yr 6	Total scheduled curricular time (min)
**1 Nijmegen**	Th on palliative care, SP in interactive lecture, 300 s/g	Th, home assignment, E-learning	Role-play +Fb. 10-15 s/g		Home assignment, Th, role-play + SP + Fb. 3 s/g.			505
**2 Rotterdam**		Th, home assignment, practice with video/DVD, role-play + Fb. 24 s/g		Th, role-play + SP + Fb. 12 s/g	Th, role-play + SP+ Fb. 4 s/g	Reflection on experience. 10-15 s/g		390
		^Timing of questionnaire about take-home messages						

**Notes:** Yr = year, Th = theory, SP = simulated patient/actor, Fb = feedback, s/g = number of students per group, BNN = breaking bad news

Grey area = clerkship in hospital

Incidental practice in, or observation of BBN during clerkships is not included in the table.

In school 1, after a non-compulsory interactive lecture about palliative care (including reference to BBN) for all students in year 1, the 2nd year students completed home-assignments followed by an e-learning on the art of breaking bad news (120 min) at the medical faculty building. The goals of the e-learning, as stated in the student’s instruction, were to learn the steps in breaking bad news, how to interpret and deal with emotional reactions of the patient, and to recognize (non-) verbal reactions of the patient. It consisted of two videos of an experienced oncologist performing an adequate and a poor BBN-conversation with a simulated patient (SP) and the subsequent effects on the patient. The videos were interrupted by online questions to be answered by the student. The used model on BBN was full/individualized disclosure following a three-phase model derived from SPIKES (break the bad news; dealing with reaction/emotions of the patient; informing about follow-up, following patient’s needs and pace (patient-centered). The e-learning concerned only major bad news (oncology). Later that week, the e-learning was followed by a small workshop on palliative care, in which a BBN conversation with a peer could be practiced if desired by the participating students. In year 4, the BBN skills training curriculum was concluded with a small group session with a SP and feedback from a surgeon and/or psychologist (described in more detail in ( van Weel-Baumgarten *et al.*, 2012)).

School 2 started in year two with self-study as a preparation for a small group session on BNN (24 students/group). The goals of this session, as stated in the student’s instruction, were to learn the steps in BBN, important do’s and don’ts and how to recognize the psychological effect on the patient after a BBN conversation. The session included watching a DVD (film fragments showing BBN in different ways) followed by discussion and peer-role-play with feedback by experienced clinicians and psychologists. In year 3 and 4 another skills training with a simulated patient was planned (small group session with 12 (yr3) and 4 (yr 4) students/group). The curriculum on BBN was concluded with a session where students reflected on their experiences in clinical practice in year 5 (max. 15 students/group). The used model on BBN was full disclosure following SPIKES with 100% major bad news ( van Weel-Baumgarten *et al.*, 2012).

### Participants and data collection

All 2nd year medical students during the years 2010-11 were invited face-to-face to join the study immediately before the start of the e-learning (school 1) and small group session (school 2). They were asked to report up to 5 take-home messages formulated in their own words (handwritten) on an open questionnaire *.* Students were given the questionnaire immediately after their training on breaking bad news after a short verbal explanation of the reason of the study: in school 1 immediately after the e-learning in year 2, in school 2 after the small group sessions with peer role-play in year 2. It was stressed that participation was voluntary and anonymous, and that their decision whether or not to participate would neither be recorded, nor affect their study progress. Students gave written informed consent. As the research involved anonymous questionnaires and participation was voluntary, the study was exempt for approval by the universities’ ethical review committees. All students agreed to participate (592 in total, 296 in each school). The researchers did not know the participants prior to the study commencement and only the participants and the researchers were present when the questionnaires were administered.

### Analysis

We analysed the data through a hybrid form of qualitative content analysis ( Hsieh and Shannon, 2005; Sandelowski, Voils and Knafl, 2009). After an open coding round on the whole dataset (round 1), the researchers (MB and AdlC) undertook a directed content analysis, for which the SPIKES mnemonic was used as a conceptual frame ( Baile *et al.*, 2000). SPIKES is a widely used model for BBN consultations which helpfully lists clear behaviours on how to BBN. In the second round, two coders (MB and AdlC) each coded parts of the data and discussed. This iterative process continued until they both coded half of all data, reaching at least 80% agreement. The two coders then each coded the whole dataset (round 3). Statements that did not fit the SPIKES framework, were inductively analysed and grouped into new codes (round 4, table 2). In round 4, the category “EMOTION” was also further divided into subcategories.

**Table 2.** Coding scheme BBN: statements included in categories

SPIKES-CATEGORY	According to BAILE (2000)	Code in our data:	Everything concerning:
S - **SETTING** up interview	Setting up the interview: incl. mental rehearsal, reviewing plan how to tell, how one will respond to reaction patient. arrange privacy involve sign others sit down make connection pt (eye contact, touching arm or holding hand) manage time constraints and interruptions	**SETTING UP**	preparation (knowing case, what do you want to say, how do you think the patient might react, how do you think you will react) attitude doctor body language doctor inviting partner, spouse, family quiet place, beeper off comments about verbal, para-verbal or non-verbal communication
P - assessing patient’s **PERCEPTION**	Assessing patient’s perception: Ask what the pt knows about illness and is it serious?	**PERCEPTION**	what does the patient know about his/her illness and is it discussed?
I - obtaining patient’s **INVITATION**	Obtaining patient’s invitation: what and how does the patient want to know?	**INVITATION**	discussion what/how much the patient wants to know about his/her illness
K - giving **KNOWLEDGE** and information to patient	Giving the medical facts. Warning the patient that bad news is coming. start at level comprehension and vocabulary pt. non-technical words avoid excessive bluntness info in small chunks check pt understanding when poor prognosis, avoid “nothing we can do”, patient might have other goal.	**INFORMATION TO PATIENT**	medical information giving hope how to give bad news (no jargon, not too blunt, small chunks, on comprehension level patient) check understanding patient being direct, straight-forward being honest
E - addressing patient’s **EMOTIONS** with empathetic responses	Responding to pt emotions: doctor offers support and solidarity by empathic response: 1. observe emotion 2. identify emotion 3. identify reason for emotion 4. give pt time to express feelings, then connecting statement (‘I know that this is not what you wanted to hear...’) Acknowledge and express own emotion as connecting statement.	EMOTIONS - REFLECTION EMOTIONS - GIVING SPACE EMOTIONS - SILENCE EMOTIONS - GENERAL	dealing with emotions (patient’s, spouse’s/family) silence taking time for emotions giving space for emotions trying to understand worries patient dealing with own emotions
S - **STRATEGY** and summary	Ask if they are ready for the discussion about treatment plan. Understand pt specific goals of treatment (e.g. symptom control)	**STRATEGY**	care after delivering bad news (walking to hallway) treatment seeing patient returns home safely
		BEING PATIENTCENTRED	listening to patient patient centered only answering questions patient not following own agenda tune in to patient
		FEEDBACK ON EDUCATION	general comment on organization or content of training BBN
		OTHER	statements that do not fit into the other categories

BBN= breaking bad news

Data were analysed with Atlas.ti version 7.1.5. We quantified the data to simplify the multitude of data and to understand the data from the sensitizing concept viewpoint by counting the frequencies of the categories identified in the analysis ( Sandelowski, Voils and Knafl, 2009).

## Results/Analysis

In total 2416 codes were assigned to the 2102 statements given, roughly equally divided between both schools. Overall, students in both schools most frequently reported take-home messages that could be grouped into 4 themes. Three of the themes concerned actual skills (how to BBN): (1) how to inform the patient, (2) how to deal with patient’s emotions, (3) how to prepare for a BBN conversation. One of the themes concerned an underlying professional quality (how to be a doctor): (4) being patient-centred, see table 3.

**Table 3.** Frequencies codes on students’ learning outcomes after BBN education.

CODES	Both schools		Nijmegen (school 1)		Rotterdam (school 2)	
	Number of codes (n)	%	Number of codes (n)	%	Number of codes (n)	%
**INFORMATION TO PATIENT**	591	24,5	244	20,2	347	28,7
**DEALING WITH EMOTIONS (total)**	499	20,6	244	20,2	255	21,1
EMOTIONS - REFLECTION	63	2,6	10	0,8	53	4,4
EMOTIONS - GIVING SPACE	78	3,2	68	5,6	10	0,8
EMOTIONS - SILENCE	152	6,3	61	5,0	91	7,5
EMOTIONS - OTHER	206	8,5	105	8,7	101	8,4
**PREPARATION**	409	16,9	224	18,5	185	15,3
**BEING PATIENT-CENTRED**	202	8,4	108	8,9	94	7,8
**FEEDBACK ON EDUCATION**	102	4,2	46	3,8	56	4,6
**STRATEGY**	80	3,3	52	4,3	28	2,3
**INVITATION**	7	0,3	0	0,0	7	0,6
**PERCEPTION**	1	0,0	0	0,0	1	0,1
**OTHER**	525	21,7	291	24,1	234	19,4
**TOTAL**	2416	100,0	1209	100,0	1207	100,0

Below we will report the results in more detail.

### How to inform the patient

The majority (24,5%) of the self-reported take-home messages were about how to tell the bad news to a patient and his/her family. This category included all statements about providing information, including medical information, the skill of how to break bad news (no use of jargon, not too blunt, small chunks of information) and considering the comprehension level and vocabulary of the patient. It also included all statements of being direct and straightforward, and honest. This is illustrated by the following quotes:

“Break bad news immediately, do not beat around the bush.” (student 153, school 1)

“Always tell the patient the truth.” (student 2.14, school 2)

*“To honestly answer patient’s questions. However, do not tell things a patient does not want to hear.”* (student 2.2, school 2)

Additionally, this category included all statements about giving hope. In the following quote a student described the importance to frame information to the situation of the patient, and to trade a delicate balance between the amount and emphasis of information provided.

“Doctors should handle a patient’s hope with care. He should be careful not to give a patient false hope, but on the other hand he should be careful that a patient does not lose all hope.” (student 12, school 1)

### How to deal with emotions

The second most frequently mentioned take-home message (20,6%) concerned ‘Dealing with emotions’ and included everything regarding dealing with the emotion of the patient and their families, but also with one’s own emotions when breaking bad news. Students mentioned different aspects of dealing with emotions: we found sub codes ‘giving space for expression of emotions’ and ‘reflection on emotions’. However, most frequently mentioned (30,4% of all emotion-statements) was ‘the use of silence’ as illustrated by the following quotes:

“To be quiet is more important than I thought.” (student 5.12, school 2)

“Be silent often, to give someone time to express emotions.” (student 9.21, school 2)

The sub code ‘Emotions-general’ concerns general statements about dealing with emotions.

### How to prepare for a BBN conversation

Take-home messages regarding how to prepare for a BBN conversation were mentioned in 16,9% of all statements. Students most commonly mentioned practical issues in preparing BBN, by using statements as ‘find a quiet room’, or ‘make sure pager is off’. Others also mentioned that the partner or family should be invited when planning a BBN conversation with the patient. Furthermore, students reported that preparation of what one wants to say, knowing the case and being up to date with the latest (medical) information, and imagining beforehand how patients might react or how they themselves would react when breaking the bad news, were also part of preparation. This is illustrated by the following:

“Think in advance about what to expect and be well informed about the patient’s medical situation.” (student 5.8, school 2)

“Think about what your own feelings and emotions regarding the illness and prognosis of the patient are before starting the bad news conversation. In this way you prepare for the eventuality that you may become emotional yourself.” (student 6.1, school 2)

Some mentioned that emotional preparation for BBN is not possible, as illustrated by the following quote:

“One cannot prepare for such a conversation, everyone reacts differently and so do you as a doctor.” (student 11.7, school 2)

Statements also included the doctor’s para-verbal (e.g. talking speed) and non-verbal behaviour (e.g. making eye contact, touching arms, holding hands), such as:

“Talk slowly, watch the patient’s attitude.” *(student 10, school 1)*

“When breaking bad news, the doctor’s attitude is essential (eye contact etc.)” *(student 179, school 1)*

“No pens, no coins in hands when breaking bad news.” *(student 6.2, school 2)*

### Being patient centred

In addition to the take-home messages that fit the practical application of BBN, students also referred to an underlying professional quality that is needed when BBN (see table 3). This quality is best described as ‘Being patient-centred’, in which the patient is regarded as the centre piece in the BBN conversation; his or her ideas, wishes and interest are being put first and the BBN conversation should be adapted to this. This is illustrated by the following quotes:

“Only react to the patient’s questions and remarks, as a doctor you should not lead the patient in the discussion.” (student 5.1, school 2)

“Always listen to the patient’s questions and do not impose your own point of view beforehand.” (student 5.8, school 2)

“Breaking bad news conversations are different for each patient. Every patient is different! Take this into consideration.” (student 2.5, school 2)

The category ‘Other’ included mostly general statements regarding the training, such as “I learned how to break bad news” without further explanation what exactly they learned, making it impossible to link the statement to one of the themes.

#### Differences between schools

When comparing the two individual schools scores on take-home messages, there were only small differences ( table 3). The largest difference was seen in the theme “How to inform the patient”, in which students from school 2 mentioned this more frequently than those from school 1 (28,7% vs. 20,1%).

Another difference was in the category “How to deal with emotions”. Students from both schools mentioned this with equal frequency (20,1% vs. 21,1%), but referred to different strategies, varying from more passive (being silent, giving space; mentioned by 10,6% in school 1 vs. 8,3% in school 2) to more active (reflection on emotions; mentioned by a 4,4% in school 2 vs. 0,8% in school 1).

## Discussion

This research is, to the best of our knowledge, the first of its kind studying medical students’ take-home messages after their first introduction and skills training on breaking bad news early in the medical curriculum. It shows us that, although the students did not have much medical experience yet, they mentioned many take-home messages that had an (emotional) impact on them reflecting knowledge about BBN, including how to prepare for and actually break the bad news and deal with the patient’s emotions. This supports the current best practice on communication skills training of starting early, so that this knowledge can further act as a basis for reiteration and reinforcement of breaking bad news skills throughout medical training (helical model) ( Kurtz, 1998; Rosenbaum, Ferguson and Lobas, 2004). Students also reported, in addition to the art of breaking bad news itself, on the underlying professional qualities needed to successfully break bad news.

Interestingly, our study shows that although the medical students are at the beginning of their medical career with no experience in BBN, they picked up many essential elements of the theory about the art of BBN after only one session. The mentioned take-home messages reflect what is on the top of their mind immediately after the first BBN training. When compared to the SPIKES-model that is frequently used for BBN education, students mostly report, as can be expected from novice learners, take-home messages particularly referring to the art of how to break the bad news itself: how to prepare for a BBN conversation, how to tell the bad news to patients, and how to deal with subsequent intense emotions.

### Domains for early BBN teaching

A number of specific domains of SPIKES were hardly mentioned by the students as take-home messages: “Assessing patient’s perception”, “Obtaining patient’s invitation” and “Strategy and summary”, relating to two of Baile’s objectives of the BBN conversation e.g. gathering information and eliciting collaboration for treatment plans ( Baile *et al.*, 2000). This may reflect the position of second year medical students, who were introduced to BBN for the first time and only had very little experience in real life contact with patients. The detailed interaction with patients and their agendas might be too complicated in this phase and not ‘just in time’ ( Kaufman, 2003) and therefore better left to later follow-up teaching. Based on this, we propose to start BBN education early and focus on preparation, information giving and the use of silence in dealing with the emotions. Based on these dimensions role models can be observed and evaluated, and it can be a basis for further development of BBN skills. Later on, in medical school, when students master these basics and gain more experience with patients, they then can evaluate their existing knowledge about BBN and adjust learning goals, as suggested by leading theories ( Taylor and Hamdy, 2013). In this way their breaking bad news skills can be broadened and refined using SPIKES and evolve into a more patient-centered, individualized disclosure and personalized way of dealing with the patient’s and one’s own emotions. This supports the need for a helical, longitudinal curriculum on breaking bad news with multiple moments to practice in a safe environment with room for trial and error.

### Learning about professionalism

Based on our observation that students often mentioned the importance to connect BBN to the ideas, emotions and wishes of the patient, we concluded that they considered ‘being patient centered’ as take-home message, in addition to learning “how to” break bad news. This is an underlying professional quality that is essential for adequately breaking bad news. The goal of communication skills training is to teach students the art of how to break bad news. However, this study shows that, although it seems somewhat early to teach BBN skills in preclinical years, students also seem to learn at a deeper personal level e.g. formulating take-home messages regarding professional qualities. This agrees with Fink’s “Taxonomy of Significant Learning” ( Fink, 2003), in which the various kinds of learning are described as synergistic, e.g. learning in one category of the taxonomy, for example the category Application, enhances learning in another category such as the Human Dimension or Care.

Furthermore, Helmich *et al*. ( Helmich *et al.*, 2014) showed that medical student’s emotions in early clinical practice influence learning outcomes. And although students in this study did not practice with real patients, it might well be that emotions emerging during this early BBN training also influenced learning outcomes. This might explain the fact that students spontaneously mentioned professional qualities such as being patient-centred, and this stresses that communication (skills) training also addresses ‘how to be a good doctor’. This also emphasizes the importance of good role models ( Paice, Heard and Moss, 2002; Lempp and Seale, 2004): not only in the clinical phase of medical school, but even in the pre-clinical years.

### Honest and/or patient-centered?

Moreover, when looking in detail into the theme How to inform the patient, statements about honesty show a remarkable dichotomy: ‘honestly telling the patient’ and ‘being honest (as a doctor)’. This might suggest that, apart from telling the truth about bad news, a good doctor also should be honest (as a professional quality).

Being honest may contradict being patient-centered, since some patients do not want to be fully informed about their illness. It is a core aspect of professionalism to deal with this tension and it is an important finding that students did mention this as a take-home message: apparently, the teaching did clarify the delicate balance between these two qualities that have to be preserved in breaking bad news.

It may also stress the importance of follow-up teaching to focus on professional qualities underpinned in the SPIKES model (‘Assessing patient’s perception of his/her illness’ and ‘Obtaining his/her invitation to reveal information’). These are both related to respecting a patient’s wish on that matter and putting the interests of the patient first. In line with what is mentioned above, these basic professional qualities are vital for an adequate BBN conversation and much needed when advancing in breaking bad news beyond just bluntly telling the bad news. An adequate BBN conversation is dependent on the ability of the doctor to weigh different factors in how and how much to tell the patient (individualized disclosure). It is promising that novice learners pick up this underlying element and that they are aware of the importance of it, as shown earlier by De Valck ( De Valck, Bensing and Bruynooghe, 2001).

As the two schools both use a different disclosure model as the basis of BBN the scores could also be interpreted in that aspect. Indeed, school 1 reported higher scores on being patient-centered than school 2 and this fits the individualized disclosure model as taught by school 1. However, the difference between school 1 and school 2 was very small. School 2 mentioned ‘being honest’ more frequently in the category ‘Information to patient’ and this might be due to the advocated full disclosure model of this school.

Since in school 2 ‘Being patient-centred’ was also frequently mentioned, it remains the question whether the formal learning goal in BBN education of full-disclosure had been adhered to or in practice the focus had been on individualized disclosure. Here the personal preference of teachers might have been more important than the formal teaching goal. Further investigation of students’ and medical professionals’ viewpoints about the relationship between honesty and patient-centeredness when breaking bad news could be valuable.

### The importance of silence

Students in both schools mention that ‘being silent’ is an important take-home message when dealing with emotions. This might be explained by the fact that it is new for intervention-prone students that the best way to deal with emotions in a conversation is not per se doing something, but rather the opposite: being silent (an allegedly passive act), which does not suit the perceived goal of the training, namely communication skills training. However, the act of ‘being silent’ by the doctor is an active act - active listening, watchful waiting - an approach that needs sophisticated communication skills (summarizing and structuring within the consultation), an approach that students pick up from their teachers ( Robertson, 2005).

### Differences between schools

When comparing the schools, both have a longitudinal, helical communication skills classroom curriculum with an early introduction to BBN, as suggested by Rosenbaum as best practice ( Rosenbaum, Ferguson and Lobas, 2004). Although the schools have a different teaching approach, the take-home messages show the same themes. This might indicate that the specific format of an introduction to BBN (either e-learning or small group session) does not influence the reported take-home messages. When looking in detail, school 2 stressed “Information to patient” and a more active approach to dealing with emotions (reflection on emotions) than school 1. Of course, this can be a matter of teachers’ semantics, yet it could also be indicative of an underlying preference of how to deal with emotions. Furthermore, both differences might be explained by the more active teaching method of school 2, in which students experience by peer role-play what it is like to break bad news, even when it is in a simulated environment, and thus focusing more on the art of ‘how to’ break bad news (How should I say it? How should I deal with emotions?) and promoting reflection on one’s behaviour. How learning methods influence learning outcomes and which teaching approach is more cost-effective is a topic of interest for further research.

### Strength and limitations

Strength of this study is that it investigated for the first-time student’s take-home messages after an introduction to BBN early in the medical studies. It also yielded an unusually high response rate, even for an anonymous data collection. Reasons for this were, in our opinion, the close connection of the data collection to the time and place of the teaching and the students’ anticipated high relevance of adequate BBN for their future career. Furthermore, based on the study aims, a worldwide influential framework (SPIKES) was used to categorize the take-home messages. This framework was not able to fit all mentioned take-home messages, and thus we had to use open coding to describe the remaining learning outcomes. This could have made it possible that the individual teacher’s favourite topic regarding breaking bad news was reflected in the mentioned messages in school 2, instead of student’s self-formulated take-home messages. It might have been possible that an open coding strategy on the whole dataset had brought different findings.

Another limitation of this study is, that we investigated the take-home messages immediately after finishing the workgroup or e-learning. We do not know, how the take-home messages change over time, when students had time to digest the new information about BBN. Furthermore, we were not able to observe student’s actual performance on BBN after this first teaching, which could have taught us more about the possible gap between ‘knows how’ and ‘shows how’. However, this study is a starting point for further investigation.

## Conclusion

This study demonstrates that students can be educated on BBN early in their curriculum and that this teaching additionally triggers learning on a personal level on professional qualities such as honesty and patient-centeredness. However, the SPIKES mnemonic for BBN seems too complicated for these novice students. Therefore, we suggest a new mnemonic PRINSE (Preparation-Information-Silence Emotions) as guideline in BBN for novice learners, which can be broadened into SPIKES in a helical curriculum approach after gaining experience in BBN. Moreover, this study stresses the importance of good role-models, not only in the clinical phase, but also in the pre-clinical years.

### Practice Implications

Based on our findings, we would like to recommend an early approach to breaking bad news teaching using the domains preparation, information giving, the use of silence in dealing with the emotions. This can be summarized in a new mnemonic: PRINSE (Preparation-INformation-Silence Emotions) as the initial focus on BBN, in the longitudinal, helical curriculum structure of communication skills teaching. This could act as a basis for further development of BBN skills by using SPIKES later. Furthermore, we would like to stress the importance of good role models, as students not only pick-up how-to BBN, but also learn underlying professional qualities such as honesty and patient-centeredness.

## Take Home Messages

• Breaking bad news training for medical students is important.
• Best practice suggests starting early following a helical curriculum.
• Our study shows that this is possible and that this teaching additionally triggers learning on a personal level (honesty and patient-centredness).
• To suit the experience of novice learners a new mnemonic PRINSE is proposed to guide this group when first learning how to BBN.
• This mnemonic can be broadened into SPIKES after gaining more clinical experience in BBN.

## Notes On Contributors

Marianne Brouwers, MD PhD, is a general practitioner and associate principal lecturer at the Department of Primary and Community Care of the Radboud University Medical Center, Nijmegen, The Netherlands. Her focus of research is the development of clinical communication skills training and assessment of medical students.

Anne de la Croix, PhD, is an assistant professor positioned at LEAN! & LEARN! Academy, Vrije Universiteit Amsterdam, and at Research in Education, VUmc School of Medical Sciences, Amsterdam, The Netherlands. Her main interests are communication & interaction in education, professional development of (future) doctors, qualitative & mixed methods.

Roland Laan is professor of Medical Education and director of Medical Education at the Radboud University Medical Centre, and head of the Radboudumc Health Academy, Nijmegen, The Netherlands.

Chris van Weel is emeritus professor of Family Medicine, Radboud University Medical Center, Nijmegen, The Netherlands and honorary professor of primary health care research, Australian National University, Canberra, Australia. He is past president of the World Organization of Family Doctors WONCA. Research expertise is in long-term outcome of patients with chronic morbidity.

Evelyn van Weel-Baumgarten MD PhD is an associate professor in medical communication, emeritus at the Department of Primary and Community care, Radboud University Medical Center, Nijmegen, the Netherlands, and president of EACH: International Association for Communication in Healthcare. Her interests include research and teaching of communication skills, including faculty development and implementation issues.
