# Supplementary material for: The impact of early breaking bad news education: a qualitative study into students' self-reported take-home messages
Source: MedEdPublish (2016). 2020 Feb 7;9:25. [Version 1] doi: 10.15694/mep.2020.000025.1 (PMC10697453; doi:10.15694/mep.2020.000025.1)
Supplement: Peer review history

# Reviewer report: Reviewer response for version 1

**DOI:** 10.21956/mep.19970.r30358

**Published:** 2020-05-15

**Authors:** Ken Masters

**Affiliations:** 1 Sultan Qaboos University

This review has been migrated. The reviewer awarded 5 stars out of 5 An interesting qualitative study on the impact of early breaking bad news education. The paper carefully explores the problems of not teaching BBN and the difficulties of doing so, and the various models used. The aim in this research was to identify novice students’ learning and reaction to an intervention.Rather than follow a simple Kirkpatrick Level 1 evaluation, the authors have used a qualitative approach. More difficult and time-consuming to implement and assess, but also more likely to get valuable and usable insight into the students’ learning and understanding. The authors have performed their tasks very well indeed. The comments from the students are gems, and I’m sure would prove invaluable to anyone teaching this subject, and also to other students. My only complaint (a little unfair, I acknowledge) is that there are too few examples of the students’ comments. I know that, for most papers, the general rule is “Theme followed by 2-3 comments”, but that is usually because of a space practically. MedEdPublish does not have that limitation. Perhaps the authors would consider creating a separate document with more of the comments, and placing it into an institutional repository, and supplying a link.It might be interesting, in a few years’ time, to run a follow-up study with these students. Unfortunately, much research shows a decline in medical student empathy, and a follow-up study would shed some (positive or negative) light on that topic.

Reviewer Expertise:

NA

No decision status is available

# Reviewer report: Reviewer response for version 1

**DOI:** 10.21956/mep.19970.r30357

**Published:** 2020-02-15

**Authors:** Ian Wilson

**Affiliations:** 1 International Medical University

This review has been migrated. The reviewer awarded 5 stars out of 5 This is an interesting study. Many would feel that introductory sessions would lead to little change in students. this study indicates it is more than that. The interesting issue is whether this information is retained and used by t he students in the real situationThank you for a well conceived and completed study.

Reviewer Expertise:

NA

No decision status is available

# Reviewer report: Reviewer response for version 1

**DOI:** 10.21956/mep.19970.r30359

**Published:** 2020-02-11

**Authors:** Virginia Randall

**Affiliations:** 1 Uniformed Services University of the Health Sciences

This review has been migrated. The reviewer awarded 4 stars out of 5 Thanks to the authors for undertaking this study. I especially appreciate their qualitative analysis technique of a modified content analysis. My take-home message was of the ability of the students to (presumably) feel empathy for the patient/family while even just thinking of BBN. Using the helical framework, it will be important to survey this group of students at some future point to explore whether and how the take-home points change.

Reviewer Expertise:

NA

No decision status is available
